# Longitudinal proteomic analysis of pathophysiology in plasma and bronchoalveolar lavage fluid of patients with ARDS

**DOI:** 10.1186/s40560-025-00793-z

**Published:** 2025-05-15

**Authors:** Yumi Mitsuyama, Hisatake Matsumoto, Fuminori Sugihara, Satoshi Fujimi, Hiroshi Ogura, Jun Oda

**Affiliations:** 1 Department of Traumatology and Acute Critical Medicine, Graduate School of Medicine, The University of Osaka, 2-15 Yamadaoka, Suita, Osaka 565-0871 Japan; 2 Division of Trauma and Surgical Critical Care, Osaka General Medical Center, 3-1-56 Bandai-Higashi, Sumiyoshi-ku, Osaka 558-8558 Japan; 3 Division of Immunology, Department of Future Medical Sciences, Kobe University Graduate School of Medicine, 7-5-1 Kusunoki-Cho, Chuo-Ku, Kobe, Hyogo 650-0017 Japan; 4 Department of Clinical Laboratory, Osaka General Medical Center, 3-1-56 Bandai-Higashi, Sumiyoshi-ku, Osaka 558-8558 Japan

**Keywords:** ARDS, Proteomics, Mass spectrometry, BALF, Plasma

## Abstract

**Background:**

Acute respiratory distress syndrome (ARDS) remains a significant clinical challenge, and its pathogenesis is not fully understood. Proteomic analyses of plasma and bronchoalveolar lavage fluid (BALF) in patients with ARDS have been performed to uncover diagnostic and prognostic markers, although previous studies have not adequately focused on longitudinal comparison of biomarkers. This study aimed to elucidate the proteomic profiles of patients with ARDS in the acute and subacute phases to better understand the pathophysiological progression of ARDS.

**Methods:**

This was a single-center, prospective, observational study of adult patients with ARDS in whom plasma and BALF samples were collected in the acute and subacute phases of ARDS and comprehensive proteins were identified and analyzed by mass spectrometry.

**Results:**

Plasma and BALF were collected from 21 ARDS patients and plasma from 24 healthy donors, from which 694 plasma proteins and 2017 BALF proteins were analyzed. Processes related to coagulation and complement commonly activated in plasma and BALF were more pronounced in the acute phase than in the subacute phase. In BALF in the acute phase, pathways related to humoral and immune responses were activated, whereas processes related to chaperones and protein folding were suppressed. IPA analysis showed that B cell receptor signaling was most activated, whereas heat shock protein 90 (HSP90) chaperone cycle, protein folding, and other pathways associated with cellular stress responses and proper protein processing were suppressed. The most activated upstream regulator was interferon gamma (IFN-γ) and the most suppressed was notch receptor 1 (NOTCH1).

**Conclusions:**

The proteomics of plasma and BALF from patients with ARDS were compared in both the acute and subacute phases. In BALF in the acute phase, humoral immunity, mainly B-cell receptor signaling, was activated, whereas the HSP90 cycle and protein folding mechanisms were inactivated.

**Supplementary Information:**

The online version contains supplementary material available at 10.1186/s40560-025-00793-z.

## Introduction

Acute respiratory distress syndrome (ARDS) is characterized by refractory hypoxemia and decreased lung compliance that develops as an acute reaction to infectious or inflammatory triggers [1]. Sepsis, pneumonia, trauma, and aspiration lung injury are the major risk factors for ARDS, and over-activation of the inflammatory response in the lungs by these factors can lead to increased alveolar capillary permeability and acute noncardiogenic pulmonary edema. The incidence of ARDS in the intensive care unit (ICU) is 10% with a mortality rate of 40% [2]. As the disease progresses, inflammation and edema are the primary pathophysiologies of lung injury in the acute phase, whereas repair and fibrosis develop in the subacute phase. Pathogenesis in the acute, or exudative, and subacute, or proliferative phases of ARDS is highly reversible, whereas in the chronic phase, i.e., the fibrotic phase, lung injury is irreversible. Significant progress has been made in ARDS research over the past decade, but we still do not have a thorough understanding of the pathogenesis of the disease [3]. As a result, no pharmacologic therapies have been shown to improve the prognosis of patients with ARDS, and treatment remains a challenge [2].

Proteomics is the comprehensive analysis of proteins translated at specific times in cells and tissues to investigate physiological and disease states, molecular mechanisms, and biomarkers for diagnosis and prognosis. Proteomic studies in ARDS patients have been conducted in plasma, bronchoalveolar lavage fluid (BALF), and lung tissue. Interleukin (IL)-10 in plasma and S100 calcium-binding protein in BALF have been reported to be associated with prognosis in ARDS patients, and proteomics in ARDS patients can help to identify diagnostic and prognostic markers and understand the pathogenesis [4–6]. Comparison by disease stage is useful in understanding the pathogenesis of ARDS as the pulmonary pathology of ARDS changes significantly as the disease progresses. However, previous proteomics studies of ARDS have rarely included biomarkers that have been compared longitudinally in the same patients and thus have been insufficiently focused on pathophysiologic changes.

Therefore, the aim of this study was to clarify the protein profiles of plasma and BALF during the acute and subacute phases of ARDS patients and to investigate the pathological progression of ARDS from a proteomic perspective.

## Materials and methods

### Study design and participants

This single-center, prospective, observational clinical study was conducted on patients with ARDS admitted to Osaka General Medical Center between April 2021 and March 2022. Eligible patients were 18 years of age or older admitted directly for ARDS or transferred from another hospital after being evaluated for admission to the ICU by a clinician. Patients who were lactating, pregnant, had a malignancy under treatment, or refused to participate in the study were excluded. The diagnosis of ARDS followed the Berlin definition [1]. All clinical and biological parameters, such as demographic characteristics, ventilation and length of hospital stay, and comorbidities were collected from the electronic medical record. Severity scores were recorded using the Acute Physiology and Chronic Health Evaluation II score (range 0–71) and the Sequential Organ Failure Assessment score (range 0–24) [7, 8]. The severity of ARDS was scored on a 3-point scale of mild, moderate, and severe [1]. Treatment details were collected within 24 h of admission and included antibiotics, antivirals, and glucocorticoid administration [9, 10]. Treatment during the hospital stay included extracorporeal membrane oxygenation and tracheostomy. This study involving humans was approved by the institutional review board of Osaka General Medical Center (approval number: 2021–002). Adult healthy donors who had no disease under treatment were recruited from the public. Written informed consent was obtained from all patients and the healthy donors. The study was conducted in accordance with the Declaration of Helsinki.

### Sample collection

Patient blood samples were collected during the acute phase of ARDS within 24 h of diagnosis and then during the subacute phase 5–8 days later. Blood samples were collected from healthy donors one at a time. Blood samples were collected in tubes containing anticoagulant (ethylenediaminetetraacetic acid) and immediately centrifuged at 3500 rpm for 5 min to separate plasma. The separated plasma was stored at − 30 °C until measurement. BALF was collected using a bronchial fiberscope during the acute phase within 24 h of the diagnosis of ARDS and during the subacute phase between 5 and 8 days later. BALF was collected from the lesion in the case of localized lesions or from the middle lobe of the right lung in the case of diffuse lesions. The bronchoalveolar lavage procedure was performed under aseptic conditions using a disposable AMBU® ASCOPE™ 4 (Ambu A/S, Ballerup, Denmark) according to the standardized method. Specifically, 3 × 20 mL of sterile saline was injected into the bronchioles, and after each injection, the maximum volume of fluid in the bronchioles (approximately 10 mL total) was collected in 50 mL sterile plastic tubes and centrifuged to separate the supernatant and sediment. The tubes were stored at -30 °C until measurement.

### Mass spectrometry methods

The method is the same as that reported in other studies previously conducted at our institution [11, 12], except that the major proteins were depleted from the plasma and BALF solution with a Proteome Purify 12 Human Serum Immunodepletion Resin kit (R&D Systems). The remaining proteins were precipitated with methanol and chloroform and dissolved in PTS (phase-transfer surfactants) solution [13]. The protein solution was reduced with dithiothreitol, alkylated with iodoacetamide, digested with trypsin, and purified on a C18 chip (GL Science Corporation, Tokyo, Japan). The trypsin-treated and purified solution was analyzed on a NanoElute nanoLC system coupled to a timsTOF Pro mass spectrometer (Bruker) using an NTCC C18 3-µm particle 15-cm column (Nikkyo Technos Co., Ltd.). The column temperature was set at 50.0 °C. The mobile phases were water with 0.1% formic acid (solvent A) and acetonitrile with 0.1% formic acid (solvent B). The digested peptides were eluted at a flow rate of 500 nL/min for 18 min with a 2–35% B gradient setting. The mass scan range was set to 300–2000 m*/z*, and the ion mobility revolution mode was set in the range of 0.85–1.30 Vs/cm^2^. Ion spray voltage was set to 1.6 kV in positive ion mode. Tandem mass spectrometry (MS/MS) spectra were acquired by automatically switching between MS and MS/MS modes. Bruker Data Analysis software was used to process the mass spectrometry data. Peptides were identified by database searches using MASCOT Server (ver. 2.7, Matrix Science Inc.). The mass tolerance of the precursor was set to 15 ppm, and that of the fragments was set to 0.05 Da. Mascot searches were performed using the Swiss-Prot database, and classification was restricted to *Homo sapiens*. Search results were quantified using Scaffold (Proteome Software, Inc.) and exported in CSV format. All LC (liquid chromatography)–MS/MS samples were prepared simultaneously and measured consecutively with one blank measurement in between to reduce batch effects. After removal of DECOY proteins in the research results, 694 proteins were used for analysis in plasma and 2017 in BALF.

### Statistical analysis

Protein count data were normalized using Integrated Differential Expression and Pathway analysis ver. 0.96 (iDEP.96; http://bioinformatics.sdstate.edu/idep, accessed on 28 December 2024) using a cutoff of at least 0.5 counts per million in one library [14]. To compare the proteins of the ARDS patients and healthy donors, principal component analysis was performed using normalized values. Limma-voom analysis was performed to search for differences in proteins in plasma from the acute and subacute phases and in BALF from the ARDS patients [15]. Volcano plot analysis was used to visualize significant changes in the expression list (VolcaNoseR; https://goedhart.shinyapps.io/VolcaNoseR/, accessed on 1 June 2024) [16]. Significance was defined as a false discovery rate < 0.2 with log2 fold change >|0.2|. To investigate enrichment analysis of the different proteins in the ARDS patients, Kyoto Encyclopedia of Genes and Genomes (KEGG) pathway and Gene Ontology (GO) analyses were performed using the web tool ShinyGO (ShinyGO 0.77; http://bioinformatics.sdstate.edu/go/, accessed on 1 June 2024). Ingenuity Pathway Analysis (IPA Spring release 2024) (QIAGEN, https://digitalinsights.qiagen.com/products-overview/discovery-insights-portfolio/analysis-and-visualization/qiagen-ipa/) was used for canonical pathway analysis (CPA) and upstream regulator analysis. CPA was performed using significantly different proteins to describe specific relationships between the proteins. We also analyzed the data by upstream regulator analysis. Adjusted *p* values were calculated by Z scores and Benjamini–Hochberg method with multiple comparison test correction. The Z score predicts the activation status of an upstream regulator by the pattern of proteins of the downstream state of that regulator, and normal pathways and upstream regulators were considered activated if the |Z score| was > 2 and p < 0.05. Among the proteins whose expression varied more in the acute phase than in the subacute phase, Venn diagram analysis was used to identify proteins that were variable in both plasma and BALF. The association of proteins with other clinical data was assessed using Spearman’s correlation coefficient. To determine the association between each protein and severity score in the acute phase and hospital mortality, receiver operating characteristic (ROC) curves were generated and area under the curve (AUC) values were compared. From the ROC curves, the optimal cutoff value was determined so that the Youden index was maximized. The cutoff values were used to classify patients into two groups and compare clinical characteristics. Continuous variables are presented as medians and interquartile ranges (IQR), and categorical variables are presented as frequencies and percentages. The Wilcoxon rank sum test was used to test continuous variables, and Fisher’s exact test was used to test nominal variables. All statistical analyses were performed using JMP Pro17 (SAS Institute Inc., Cary, NC, USA) and Prism 9 (GraphPad Software, San Diego, CA, USA).

## Results

### Patient characteristics

This study included 21 ARDS patients and 24 healthy donors (Table 1). The median age of the patients was 59 (IQR: 47–73) years and was not significantly different from that of the healthy donors. The median body mass index of the patients was 26.9 (IQR: 24.5–31.2) kg/m^2^, which was significantly higher than that of the healthy donors. Viral pneumonia (Coronavirus disease 2019: COVID-19) was the most common cause of ARDS, followed by bacterial pneumonia. Glucocorticoids were administered in 95.2% of patients, and extracorporeal membrane oxygenation was conducted in 33.3% of them. The median length of mechanical ventilation was 12 (IQR: 7–25) days, and the median length of stay in hospital was 27 (IQR: 16–36) days. Hospital mortality was 28.6%.

**Table 1:**
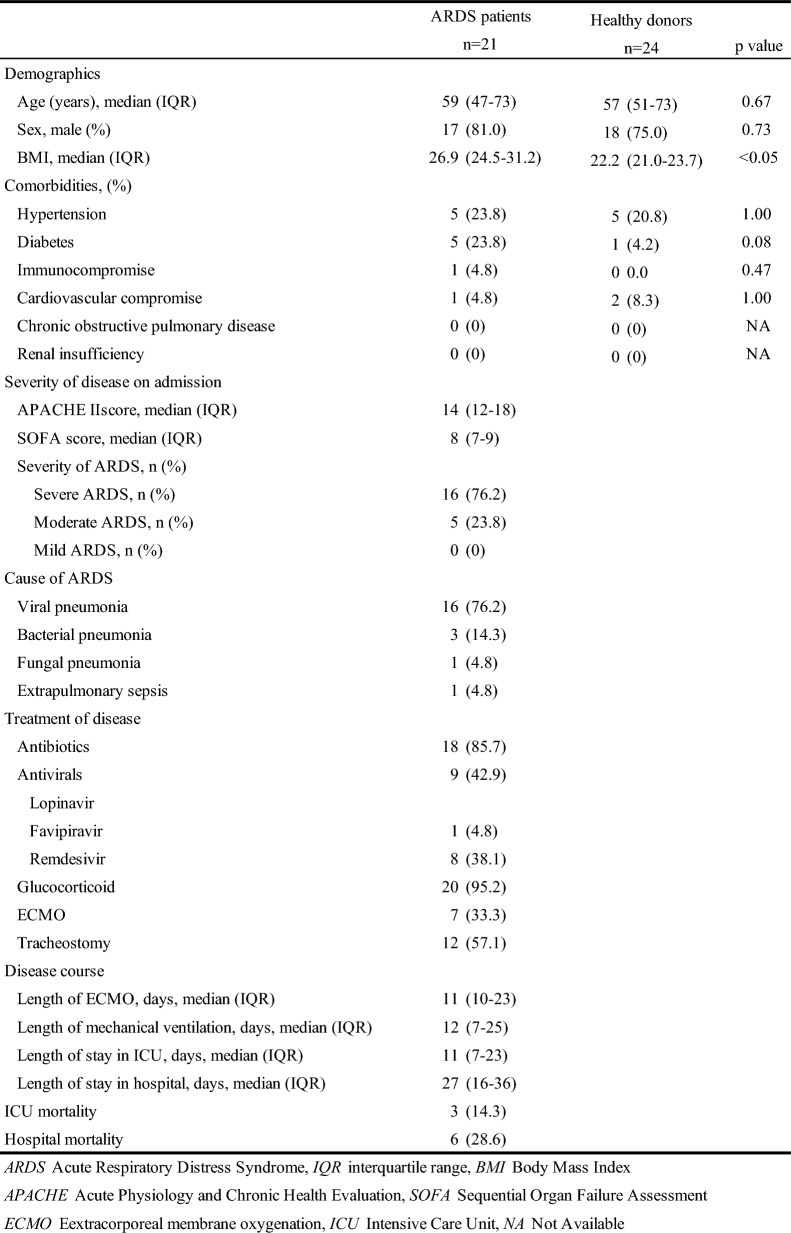
Characteristics of the population

### Analysis of plasma proteins

All of the patients had received antibiotics, antivirals, or glucocorticoid prior to collection of acute plasma and BALF. Principal component analysis of plasma proteins from the ARDS patients and healthy donors clearly distinguished between the two groups (Fig. S1). Volcano plot analysis was performed to visualize the differences in protein expression between patients in the acute phase and healthy donors. Compared to those in the healthy donors, 373 proteins were significantly more highly expressed in the patients, and 83 proteins were less highly expressed. To determine which biological processes were affected, we performed a significant protein enrichment analysis based on the KEGG and GO biological processes. Both KEGG and biological process-based pathways activated processes related to blood coagulation and complement. Processes related to intermediate filaments were inactivated in the biological process-based analysis. In addition to processes related to blood coagulation and complement in the comparison between patients in the subacute phase and healthy donors, processes related to acquired immunity and stress responses were activated in the biological process-based analysis (Fig. S2). Next, we compared the plasma proteins in the acute and subacute phases. Principal component analysis of plasma proteins from the ARDS patients in the acute and subacute phases is shown in Fig. S3. In the volcano plot, 120 proteins were significantly more highly expressed in the acute phase than in the subacute phase, and 15 proteins were less highly expressed (Fig. S3). Enrichment analysis showed that the processes related to platelet activation were those most activated in both KEGG and GO.

### Analysis of BALF proteins

**Fig. 1:**
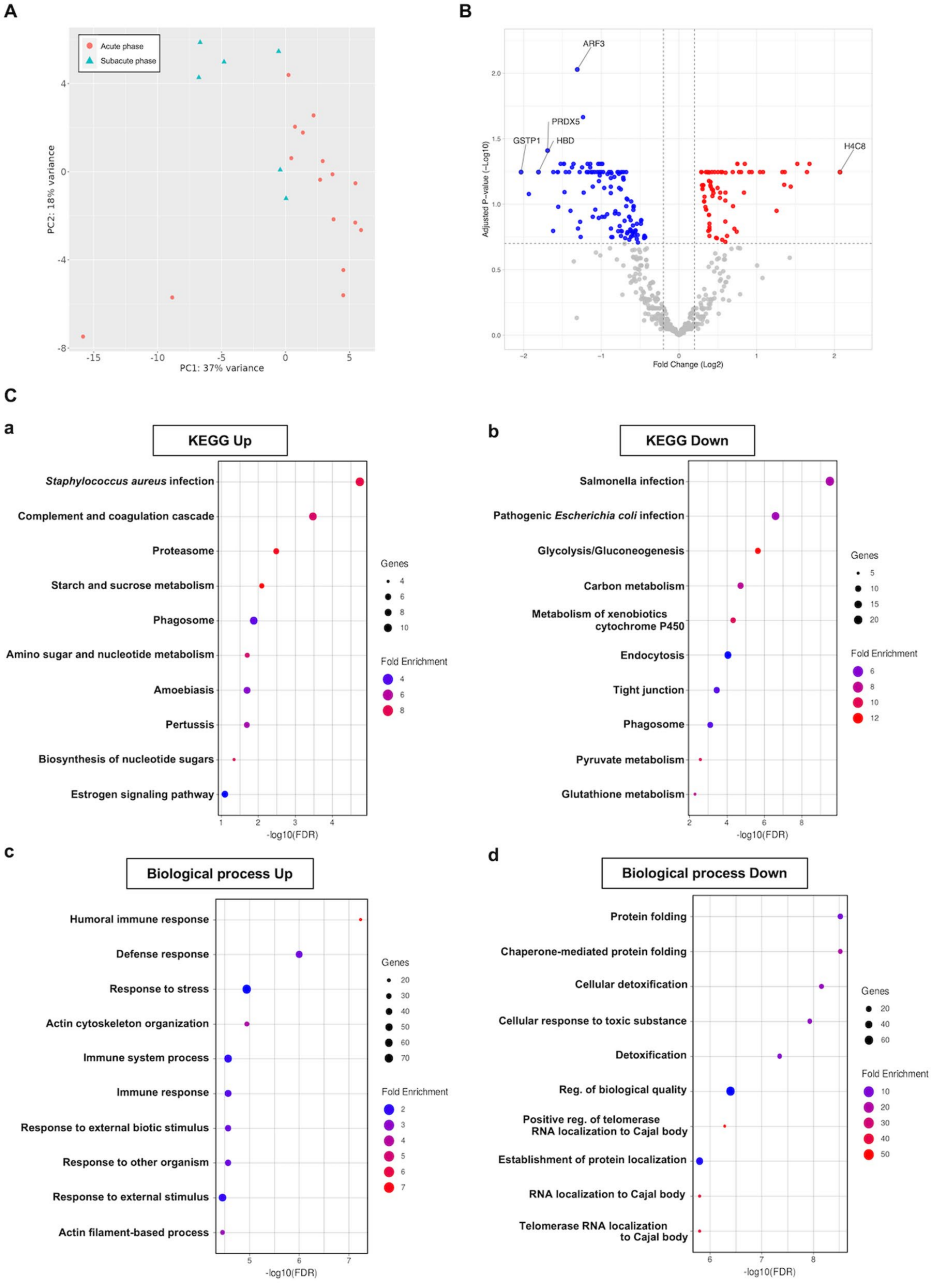
Comparison between acute- and subacute-phase BALF of patients with ARDS. **A** Principal component analysis of BALF from ARDS patients in the acute phase compared to that of the subacute phase. Red dots indicate the acute phase and light green triangles indicate the subacute phase. **B** Volcano plot of the difference in protein expression in the acute- and subacute-phase BALF of ARDS patients. The vertical dashed lines indicate |log2 fold change|> 0.2. The horizontal dashed line indicates the threshold for FDR < 0.2. Red dots indicate proteins whose expression increased, and blue dots indicate proteins whose expression decreased. The top 5 proteins with significantly different expression are shown. *ARF3* ADP-ribosylation factor 3, *H4C8* H4 Clustered Histone 8, *PRDX5* Peroxiredoxin-5, *HBD* Hemoglobin Subunit Delta, *GSTP1* Glutathione S-Transferase Pi 1. **C** Enrichment analysis based on biological processes and KEGG data. Fold enrichment is defined as in this figure. The size of the dots indicates the number of genes in the pathway. (a and c) Analyses are based on significantly up-regulated proteins. (b and d) Analyses are based on significantly down-regulated proteins. *ARDS* acute respiratory distress syndrome, *BALF* bronchoalveolar lavage fluid, *FDR* false discovery rate, *KEGG* Kyoto Encyclopedia of Genes and Genomes, *reg.* regulation

Principal component analysis of proteins in the acute- and subacute-phase BALF of the ARDS patients showed that clusters were formed, although there was some overlap (Fig. 1A). Volcano plot analysis that was performed to visualize the differences in expression of each protein showed that 186 proteins were significantly more highly expressed in the acute phase compared to the subacute phase, whereas 159 proteins were less highly expressed (Fig. 1B). The top proteins that were significantly variable in expression were different from the top plasma proteins. In the KEGG-based pathways of enrichment analysis, processes related to complement and coagulation were activated. In pathways based on biological processes, pathways related to humoral immune responses and immune responses were activated, whereas processes related to protein processing, such as chaperones and protein folding, and to detoxification were inactivated (Fig. 1C).

### IPA analysis of BAL proteins

**Fig. 2:**
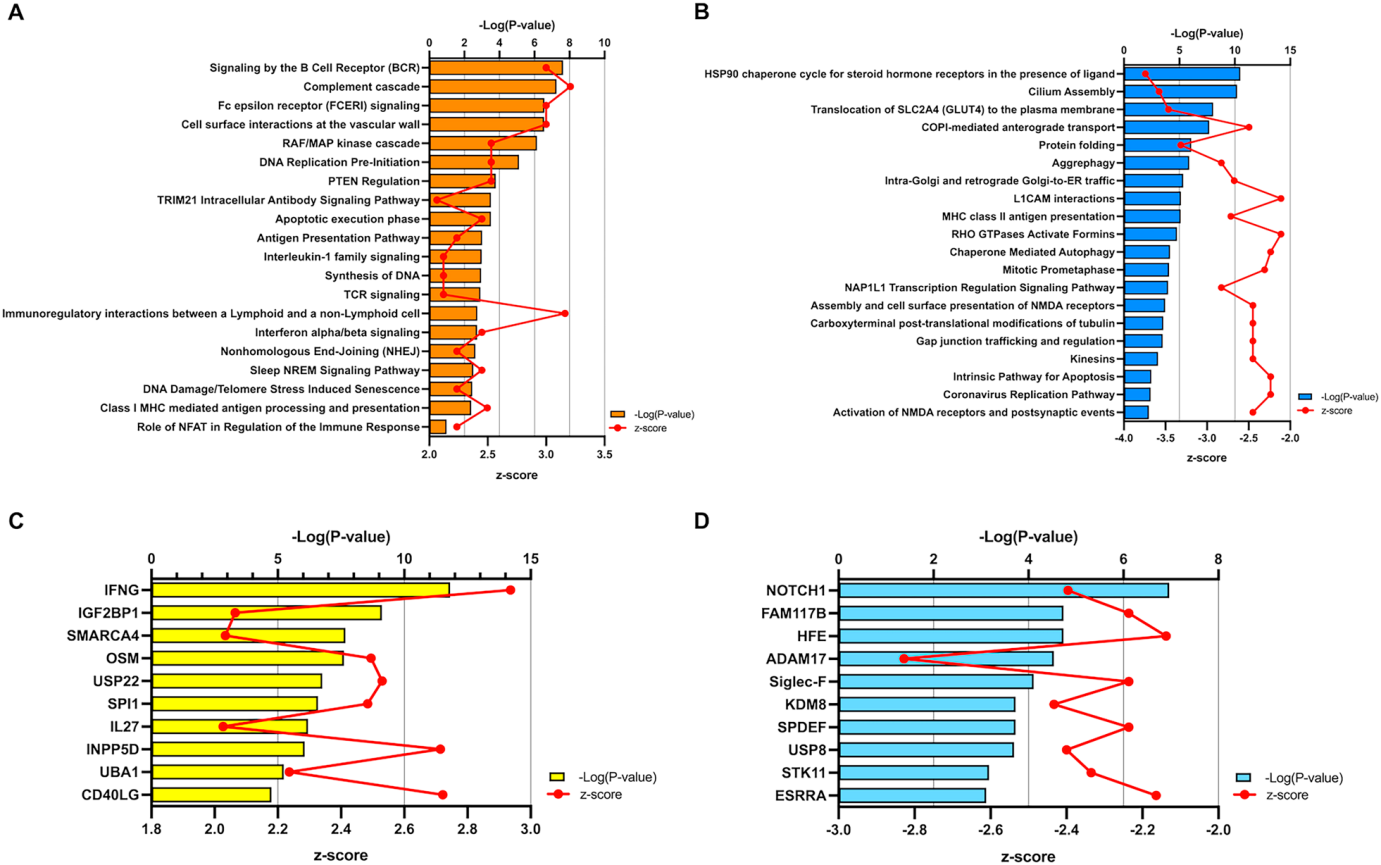
CPA and upstream regulator analysis of BALF. **A** IPA was used to identify the top 20 activated CPAs with *p* values < 0.05 for proteins in the BALF of ARDS patients. **B** Using IPA, we identified the top 20 suppressed CPAs with *p* values < 0.05 for proteins in the BALF of ARDS patients. **C** Top activated upstream regulators of proteins in the BALF of ARDS patients. **D** Top suppressed upstream regulators of proteins in the BALF of ARDS patients. *ARDS* acute respiratory distress syndrome, *BALF* bronchoalveolar lavage fluid, *CPA* canonical pathway analysis, *IPA* Ingenuity Pathway Analysis

The results of BAL mass spectrometry were submitted to CPA by IPA to list canonical signaling pathways activated in ARDS in the acute phase compared to those in the subacute phase. CPA predicted that 19 protein pathways were activated and that 22 protein pathways were inhibited (|Z score|> 2; *p* value of overlap < 0.05) (Table S1). The top 20 pathways with *p* values < 0.05 are shown in Fig. 2. According to changes in protein levels, signaling by B-cell receptor was the most activated in these analyses. There was also activation of inflammatory and immune responses, such as the complement cascade, Fc epsilon receptor signaling, IL-1 family signaling and T-cell receptor signaling. Cell surface interactions in the vessel wall and immunomodulatory interactions between lymphoid and non-lymphoid cells were activated, as were pathways related to DNA repair and stress responses (Fig. 2A). Pathways involved in cellular stress response and proper protein folding, such as heat shock protein (HSP)90 (HSP90) chaperone cycle, aggrephagy, chaperone-mediated autophagy, and protein folding, were inactivated. Intracellular trafficking and structure-maintaining processes such as cilium assembly, coat protein complex I (COPI) transport, and Golgi-endoplasmic reticulum transport were suppressed. MHC class II antigen presentation was inactivated (Fig. 2B). Upstream regulator analysis identified 29 activated and 12 inhibited upstream regulators in the BALF of the acute ARDS patients (overlapping *p* values < 0.05). These top 10 upstream regulators are shown in Fig. 2C, D. The most activated upstream regulator was interferon gamma, whereas the most inhibited upstream regulator was notch receptor 1 (NOTCH1).

### Relation to other clinical data

**Fig. 3:**
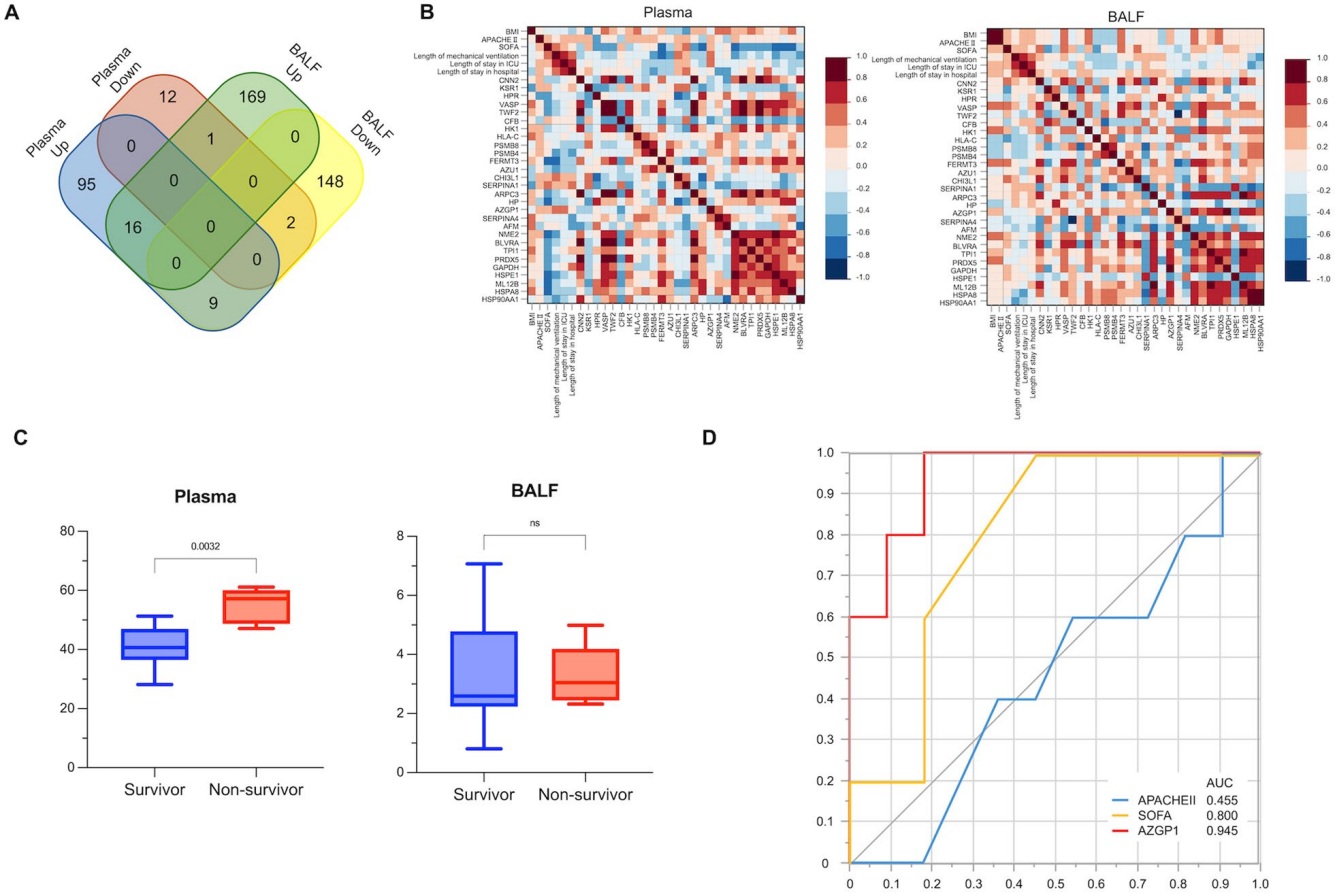
Characteristics of each protein. **A** Venn diagram analysis of proteins that differed significantly in the acute phase compared to the subacute phase is shown. Twenty-eight proteins were common between plasma and BALF. **B** Correlation chart between each protein in the acute phase and clinical information using Spearman’s correlation coefficient. **C** Comparison of AZGP1 in the acute plasma and BALF between survivors and non-survivors. All vertical axis values for each protein are exponentially modified protein abundance index values. **D** ROC curve analysis using SOFA, APACHE II, and AZGP1. AUCs were calculated and the prognostic ability of each value for hospital mortality was evaluated. *BALF* bronchoalveolar lavage fluid, *AZGP1* Zinc-alpha2-glycoprotein, *ROC* receiver operating characteristic, *SOFA* Sequential Organ Failure Assessment score, *APACHEII* Acute Physiologic Assessment and Chronic Health Evaluation II, *AUC* area under the curve, *BMI* body mass index, *CNN2* Calponin 2, *KSR1* Kinase Suppressor of Ras 1, HPR Haptoglobin-Related Protein, *VASP* Vasodilator-Stimulated Phosphoprotein, *TWF2* Twinfilin Actin Binding Protein 2, *CFB* Complement Factor B, *HK1* Hexokinase 1, *HLA-C* Major Histocompatibility Complex, Class I, C, *PSMB8* Proteasome Subunit Beta 8, *PSMB4* Proteasome Subunit Beta 4, *FERMT3* Fermitin Family Member 3, *AZU1* Azurocidin 1, *CHI3L1* Chitinase 3-Like 1, *SERPINA1* Serpin Family A Member 1, *ARPC3* Actin-Related Protein 2/3 Complex Subunit 3, *HP* Haptoglobin, *AZGP1* Alpha-2-Glycoprotein 1, Zinc-Binding, *SERPINA4* Serpin Family A Member 4, *AFM* Afamin, *NME2* Nucleoside Diphosphate Kinase B, *BLVRA* Biliverdin Reductase A, *TPI1* Triosephosphate Isomerase 1, *PRDX5* Peroxiredoxin 5, *GAPDH* Glyceraldehyde-3-Phosphate Dehydrogenase, *HSPE1* Heat Shock Protein Family E (Hsp10) Member 1, *ML12B* Myosin Light Chain 12B, *HSPA8* Heat Shock Protein Family A (Hsp70) Member 8, *HSP90AA1* Heat Shock Protein 90 Alpha Family Class A Member 1

From the proteins that differed significantly in plasma and BALF in the acute and subacute phases, respectively, proteins common to both plasma and BALF were extracted (Table S2, Fig. 3A). Sixteen proteins were elevated in both plasma and BALF, and two proteins were decreased in both. The relationship between these proteins and clinical data is shown using Spearman’s correlation coefficient (Fig. 3B). In the acute phase, plasma Chitinase 3-like 1 (CHI3L1), Serpin Family A Member (SERPINA1), and zinc-α2-glycoprotein (AZGP1) were positively correlated with severity score, length of mechanical ventilation, length of stay in the ICU, and length of hospital stay. In BALF in the acute phase, CHI3L1 and SERPINA1 showed weak positive correlations with clinical data. Of these proteins that differed significantly between the acute and subacute phases, AZGP1 was decreased in both plasma and BALF. Plasma AZGP1 was significantly increased in non-survivors compared to survivors (Figs. 3C, S4, and S5). The prognostic value of plasma AZGP1 in the acute phase was examined by ROC analysis and compared with other severity scores (Fig. 3D). The AUC was highest for AZGP1 at 0.945. Clinical information was compared between the groups with high and low plasma AZGP1 based on a cutoff value of the maximum Youden index. No differences were observed in severity of illness, shock, or length of mechanical ventilation between the two groups. In the high AZGP1 group, there was a trend toward a higher number of days on mechanical ventilation and significantly shorter survival (Fig. S6).

## Discussion

Protein changes in plasma showed activation of pathways related to coagulation and complement in both the acute and subacute phases, consistent with previous reports [17, 18]. Activation of coagulation and the complement system was more pronounced in the acute phase than in the subacute phase. Serum amyloid A1 (SAA1) and serum amyloid A2 (SAA2), which were significantly upregulated in both the acute and subacute phases, have been reported to be severity-related biomarkers in ARDS due to COVID-19 [19, 20]. Meanwhile, the SAA family is also known as a marker of obesity-related inflammatory responses as it is overproduced in adipose tissue and causes insulin resistance [21]. The high levels of SAA family members in plasma in the present study may be due to some effect of high body mass index levels in addition to the acute elevation due to ARDS. This study showed that plasma AZGP1 in the acute phase was significantly higher in non-survivors and correlated positively with patient outcome. S100 family calcium-binding protein and IL-10, which are associated with inflammation, have been reported as prognostic biomarkers of ARDS [4]. S100A8/A9 promotes inflammation and neutrophil recruitment to the lungs, thus contributing to pulmonary edema and increasing the severity of ARDS [22]. IL-10 is an anti-inflammatory cytokine with immunomodulatory activity, and low levels have been reported to be associated with poor prognosis [23, 24]. AZGP1 is an important protein mainly involved in lipid metabolism and is also known to have immunomodulatory functions [25, 26]. AZGP1 has been reported to function as an RNA-binding protein that inhibits epithelial cell proliferation in the lung tissue of patients with chronic obstructive pulmonary disease, and its deficiency contributes to disease exacerbation [27, 28]. Although plasma AZGP1 is significantly associated with mortality, the lack of significant differences in severity between the groups with low and high plasma AZGP1 possibly indicates that AZGP1 might reflect a different pathophysiology from conventional severity indices. This may be a useful additional biomarker in that it can supplement a patient’s risk that is not captured by existing scores. In addition, plasma AZGP1 may be a marker for delayed recovery from ARDS as the AZGP1 high group had higher numbers of both days on mechanical ventilation and deaths after the subacute phase.

With regard to protein changes in BALF, while the activation of complement and coagulation is common to plasma, there were several differences. The first is the presence of an excessive inflammatory immune response. Activated regulators such as interferon gamma, oncostatin M, IL-27, and CD40 ligand suggest activation of inflammatory cytokines and immune responses [29–31]. Inactivation of sialic acid binding Ig-like lectin F, NOTCH1, and ADAM metallopeptidase domain 17 suggests downregulation of inflammatory suppressive pathways and dysregulation of certain immune cells, such as eosinophils [32–34]. The coordination of the complement and coagulation cascades triggered by proinflammatory cytokines in BALF, which over-activate the immune response via signaling of B-cell receptor, T-cell receptors, and Fc epsilon receptor, amplifies excessive inflammation in lung tissue, leading to thrombotic inflammation and lung endothelial damage, which in turn contributes to the progression of ARDS [35]. Many of the patients in this study had COVID-19, and the crosstalk between inflammation and coagulation in the alveolar space is consistent with previous reports. In particular, immunothrombosis caused by crosstalk between inflammation and coagulation leads to worsening of the condition [36].

Second, mechanisms involved in the cellular stress response and proper protein folding were impaired in BALF in the acute phase. HSPs act as chaperones that prevent protein misfolding in the presence of stressors and are known to have diverse functions, such as suppressing apoptosis, stabilizing the cytoskeleton, and supporting hormone regulation [37]. In addition to these functions, the HSP90 cycle, the pathway most suppressed in the study, is known to stabilize the NF-κB signaling pathway, regulate the production of inflammatory cytokines, support mitochondrial function, and contribute to adaptation to oxidative stress [38, 39]. Dysfunction of HSP90 results in an inability to suppress the excessive inflammatory response, leading to progressive lung injury. Suppression of the protein folding process involving HSPs impairs the originally normal stress response and produces oxidative stress. Furthermore, suppression of the HSP90 cycle reduces the stress response and promotes cell death and tissue damage in inflammatory environments [39]. In addition, protein levels of the antioxidant enzyme peroxiredoxin 5 (PRDX5) and the detoxification enzyme glutathione S-transferase Pi 1 (GSTP1) are reduced in BALF, which may amplify oxidative stress and advance acute lung injury [40, 41]. Weiss et al. reported impaired pulmonary HSP expression and increased pulmonary vascular permeability in sepsis-induced rats [42]. In total, suppression of the HSP90 cycle can lead to lung tissue damage due to dysregulation of inflammation in the alveolar space and accumulation of oxidative stress.

Third, signaling by the B-cell receptor was most activated in BALF, showing activation of humoral immunity mainly by B-cell receptor signaling. Although proteomic studies of BALF in ARDS patients have reported activation of immune response processes by leukocytes and lymphocytes, it is interesting that the present study showed activation of processes by humoral immunity, mainly B-cell receptor signaling [43, 44]. Oncostatin M is known to be associated with B-cell colonization and activation of the airways [45]. Many of the study patients had direct ARDS caused by COVID-19. Direct lung injury primarily affects the alveolar epithelium and is associated with a localized alveolar inflammatory response. In contrast, indirect lung injury caused by trauma, pancreatitis, or sepsis is known to affect the alveolar vascular endothelium through inflammatory mediators in the bloodstream and shows more severe endothelial damage [46]. Yue et al. compared the proteomics of BALF in direct and indirect lung injury in lipopolysaccharide-induced mice [46]. In indirect lung injury, the Liver X Receptor–Retinoid X Receptor (LXR–RXR) activation pathway, nitric oxide and reactive oxygen species production in macrophages, IL-7 signaling, and C–X–C motif chemokine ligand (Cxcl-15) expression were all suppressed, indicating that systemic inflammation suppresses lung innate immunity in lipopolysaccharide-induced indirect lung injury. The activation of humoral immunity in BALF in the acute phase followed by activation of the same process in plasma in the subacute phase may reflect the fact that many patients had direct damage to the lungs themselves and that the immune response spilled over from the lung, the primary site of inflammation, to the whole body.

Finally, the activation of humoral immunity and inactivation of the stress response and protein folding pathway in BALF during the acute phase may be influenced by glucocorticoid administration. Glucocorticoid affects the organism primarily through immunosuppression and modulation of the stress response. Glucocorticoids are known to inhibit B-cell maturation and activation and decrease antibody production, and in the subacute phase, steroid administration may suppress humoral immune activity [47]. The glucocorticoid receptor is stabilized by forming a complex with chaperones, such as HSP90 and HSP70, and binding of glucocorticoid to the receptor dissociates the chaperone complex and temporarily impairs chaperone function [48]. In this study, glucocorticoid was administered prior to sample collection, which may have altered chaperone function. In addition, alveolar epithelial cells normally adapt to endoplasmic reticulum stress, but under conditions of excessive inflammation, the proteasome system may be overactive, leading to increased chaperone degradation and disruption of the normal unfolded protein response [49].

## Limitations

This study has several limitations. First, this is a preliminary study based on measurements of only 21 patients at a single institution. Several enrichment analyses did not detect an adequate number of pathways for discussion. Second, although we detected a large number of proteins using mass spectrometry, airway and bronchoscope contamination should always be considered. We used sterile disposable bronchoscopes to minimize contamination. Third, this study compared patients with ARDS exclusively with healthy donors, without including other critically ill patients, such as those with severe pneumonia or mechanically ventilated patients without ARDS. As a result, it remains uncertain whether the observed findings are specific to the pathophysiology of ARDS. Finally, 76.2% of the ARDS cases in our cohort were attributable to COVID-19-associated viral pneumonia. Given that immune responses may vary depending on the underlying pathogen, pathogen-specific analyses were not feasible due to the limited sample size. Therefore, our findings may predominantly reflect immune characteristics associated with COVID-19-related ARDS. Future large-scale studies that include comparisons with other critically ill populations, such as patients with severe pneumonia, are warranted to validate our results.

## Conclusion

We compared the proteomics of plasma and BALF in the acute and subacute phases of ARDS to evaluate the processes involved in its pathogenesis. Processes related to coagulation and complement were activated in both plasma and BALF in the acute phase compared to the subacute phase. In the acute phase, BALF showed an excessive inflammatory immune response and activation of humoral immunity, mainly B-cell receptor signaling, whereas the stress response and protein folding mechanisms were inactivated.

## Supplementary Information

Additional file 1: Figure S1. Comparison of acute-phase plasma of ARDS patients with that of healthy donors. **A** Principal component analysis of plasma from ARDS patients in the acute phase compared to plasma from healthy donors. Red dots indicate patients and light green triangles indicate the healthy donors. **B** Volcano plot of differences in plasma protein expression in ARDS patients and healthy donors. The vertical dashed lines indicate a |log2 fold change|> 0.2. The horizontal dashed line indicates the threshold for FDR < 0.2. Red dots indicate proteins with increased expression, and blue dots indicate proteins with decreased expression. The top 5 proteins with significantly different expression are shown. *SAA1* Serum amyloid A1, *SAA2* Serum amyloid A2, *LCP1* Lymphocyte cytosolic protein 1, *ORM2* Orosomucoid 2, *TKT* Transketolase. **C** Enrichment analysis based on biological processes and KEGG data. Fold enrichment is defined as the percentage of genes belonging to a pathway divided by the corresponding percentage of background. The size of the dots indicates the number of genes in the pathway. a and c Analyses are based on significantly up-regulated proteins. b and d Analyses are based on significantly down-regulated proteins. *ARDS* acute respiratory distress syndrome, *ECM* extracellular matrix, *FDR* false discovery rate, *KEGG* Kyoto Encyclopedia of Genes and Genomes, *reg*. regulation.

Additional file 2: Figure S2. Comparison of subacute-phase plasma of ARDS patients with that of healthy donors. **A** Principal component analysis of plasma from ARDS patients in the subacute phase compared to plasma from healthy donors. Red dots indicate patients and light green triangles indicate the healthy donors. **B** Volcano plot of differences in plasma protein expression in the ARDS patients and healthy donors. The vertical dashed lines indicate a |log2 fold change|> 0.2. The horizontal dashed line indicates the threshold for FDR < 0.2. Red dots indicate proteins with increased expression, and blue dots indicate proteins with decreased expression. The top 5 proteins with significantly different expression are shown. *SAA1* Serum amyloid A1, *SAA2* Serum amyloid A2, *LCP1* Lymphocyte cytosolic protein 1, *ORM2* Orosomucoid 2, *TKT* Transketolase. **C** Enrichment analysis based on biological processes and KEGG data. Fold enrichment is defined as the percentage of genes belonging to a pathway divided by the corresponding percentage of background. The size of the dots indicates the number of genes in the pathway. a and c Analyses are based on significantly up-regulated proteins. b and d Analyses are based on significantly down-regulated proteins. *ARDS* acute respiratory distress syndrome, *ECM* extracellular matrix, *FDR* false discovery rate, *KEGG* Kyoto Encyclopedia of Genes and Genomes, *reg.* regulation.

Additional file 3: Figure S3. Comparison between acute and subacute plasma of patients with ARDS. **A** Principal component analysis of plasma from ARDS patients in the acute phase compared to that of the subacute phase. Red dots indicate patients and light green triangles indicate the healthy donors. **B** Volcano plot of the difference in plasma protein expression between acute and subacute plasma of ARDS patients. The vertical dashed lines indicate |log2 fold change|> 0.2. The horizontal dashed line indicates the threshold for FDR < 0.2. Red dots indicate proteins whose expression increased, and blue dots indicate proteins whose expression decreased. The top 5 proteins with significantly different expression are shown. *SAA1* Serum amyloid A1, *SAA2* Serum amyloid A2, *FGL1* Fibrinogen-like protein 1, *MAN1A1* Mannosidase Alpha Class 1A Member 1, *TTR* Transthyretin. **C** Enrichment analysis based on biological processes and KEGG data. Fold enrichment is defined as the percentage of genes belonging to a pathway divided by the corresponding percentage of background. The size of the dots indicates the number of genes in the pathway. a and c Analyses are based on significantly up-regulated proteins. b and d Analyses are based on significantly down-regulated proteins. *ARDS* acute respiratory distress syndrome, *ECM* extracellular matrix, *FDR* false discovery rate, *KEGG* Kyoto Encyclopedia of Genes and Genomes, *reg.* regulation.

Additional file 4: Figure S4. Comparison of each protein between survivors and non-survivors in acute-phase plasma. All values on the vertical axis for each protein are exponentially modified protein abundance index values. *CNN2* Calponin 2, *KSR1* Kinase Suppressor of Ras 1, *HPR* Haptoglobin-Related Protein, *VASP* Vasodilator-Stimulated Phosphoprotein, *TWF2* Twinfilin Actin Binding Protein 2, *CFB* Complement Factor B, *HK1* Hexokinase 1, *HLA-C* Major Histocompatibility Complex, Class I, C, *PSMB8* Proteasome Subunit Beta 8, *PSMB4* Proteasome Subunit Beta 4, *FERMT3* Fermitin Family Member 3, *AZU1* Azurocidin 1, *CHI3L1* Chitinase 3-Like 1, *SERPINA1* Serpin Family A Member 1, *ARPC3* Actin-Related Protein 2/3 Complex Subunit 3, *HP* Haptoglobin, *AZGP1* Alpha-2-Glycoprotein 1, Zinc-Binding, *SERPINA4* Serpin Family A Member 4, *AFM* Afamin, *NME2* Nucleoside Diphosphate Kinase B, *BLVRA* Biliverdin Reductase A, *TPI1* Triosephosphate Isomerase 1, *PRDX5* Peroxiredoxin 5, *GAPDH* Glyceraldehyde-3-Phosphate Dehydrogenase, *HSPE1* Heat Shock Protein Family E (Hsp10) Member 1, *ML12B* Myosin Light Chain 12B, *HSPA8* Heat Shock Protein Family A (Hsp70) Member 8, *HSP90AA1* Heat Shock Protein 90 Alpha Family Class A Member 1.

Additional file 5: Figure S5. Comparison of each protein between survivors and non-survivors in acute-phase BALF. All values on the vertical axis for each protein are exponentially modified protein abundance index values. *BALF* bronchoalveolar lavage fluid, *CNN2* Calponin 2, *KSR1* Kinase Suppressor of Ras 1, *HPR* Haptoglobin-Related Protein, *VASP* Vasodilator-Stimulated Phosphoprotein, *TWF2* Twinfilin Actin Binding Protein 2, *CFB* Complement Factor B, *HK1* Hexokinase 1, *HLA*-C Major Histocompatibility Complex, Class I, C, *PSMB8* Proteasome Subunit Beta 8, *PSMB4* Proteasome Subunit Beta 4, *FERMT3* Fermitin Family Member 3, *AZU1* Azurocidin 1, *CHI3L1* Chitinase 3-Like 1, *SERPINA1* Serpin Family A Member 1, *ARPC3* Actin-Related Protein 2/3 Complex Subunit 3, *HP* Haptoglobin, *AZGP1* Alpha-2-Glycoprotein 1, Zinc-Binding, *SERPINA4* Serpin Family A Member 4, *AFM* Afamin, NME2 Nucleoside Diphosphate Kinase B, *BLVRA* Biliverdin Reductase A, *TPI1* Triosephosphate Isomerase 1, *PRDX5* Peroxiredoxin 5, *GAPDH* Glyceraldehyde-3-Phosphate Dehydrogenase, *HSPE1* Heat Shock Protein Family E (Hsp10) Member 1, *ML12B* Myosin Light Chain 12B, *HSPA8* Heat Shock Protein Family A (Hsp70) Member 8, *HSP90AA1* Heat Shock Protein 90 Alpha Family Class A Member 1.

Additional file 6: Figure S6. Comparison of clinical information in the low and high plasma AZGP1 groups. **A** Comparison of clinical information. Patients were divided into two groups using the plasma AZGP1 value at which the Youden index was maximal as the cutoff. *APACHE* Acute Physiology and Chronic Health Evaluation II, *SOFA* Sequential Organ Failure Assessment, *P/F* partial pressure of arterial oxygen/fraction of inspired oxygen. **B** The Kaplan–Meier curves for the two groups. The vertical axis shows the cumulative probability of survival, and the horizontal axis shows the days from admission to death.

Additional file 7.

## Data Availability

The mass spectrometry proteomics data have been deposited to the ProteomeXchange Consortium via the PRIDE partner repository with the data set identifier PXD055757.

## Abbreviations

ARDS: Acute respiratory distress syndrome
ICU: Intensive care unit
MS/MS: Tandem mass spectrometry
BALF: Bronchoalveolar lavage fluid
KEGG: Kyoto Encyclopedia of Genes and Genomes
GO: Gene Ontology
IPA: Ingenuity pathway analysis
CPA: Canonical pathway analysis
ROC: Receiver operating characteristic
AUC: Area under the curve
IQR: Interquartile range
COVID-19: Coronavirus disease 2019
HSP: Heat shock protein
AZGP1: Zinc-alpha2-glycoprotein
NOTCH1: Notch receptor 1
