# Supplementary figures and images for: Longitudinal proteomic analysis of pathophysiology in plasma and bronchoalveolar lavage fluid of patients with ARDS
Source: J Intensive Care. 2025 May 15;13:26. doi: 10.1186/s40560-025-00793-z (PMC12079806; doi:10.1186/s40560-025-00793-z)

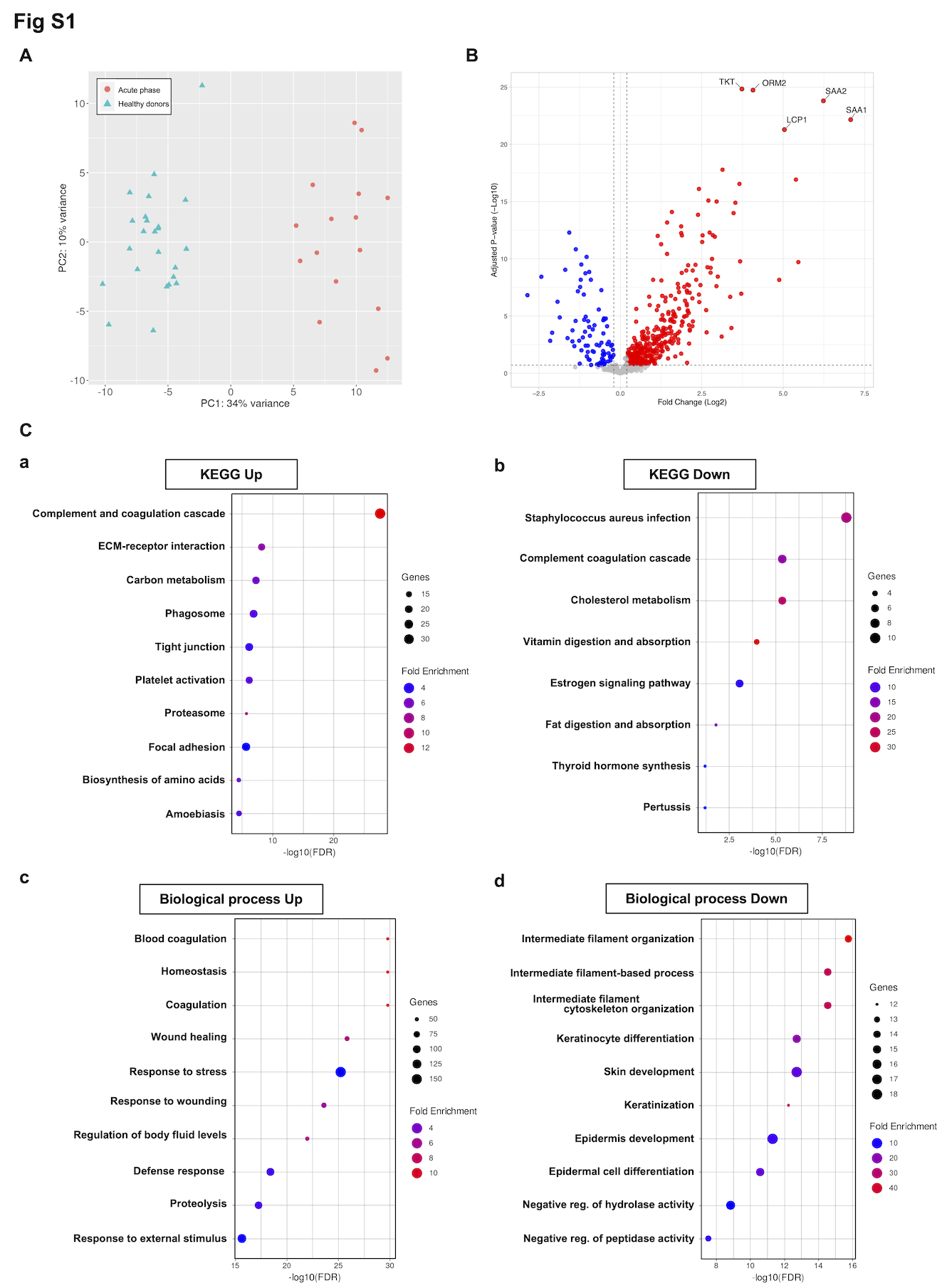

Supplement: Supplementary file 1 — Additional file 1: Figure S1. Comparison of acute-phase plasma of ARDS patients with that of healthy donors. A Principal component analysis of plasma from ARDS patients in the acute phase compared to plasma from healthy donors. Red dots indicate patients and light green triangles indicate the healthy donors. B Volcano plot of differences in plasma protein expression in ARDS patients and healthy donors. The vertical dashed lines indicate a |log2 fold change|> 0.2. The horizontal dashed line indicates the threshold for FDR < 0.2. Red dots indicate proteins with increased expression, and blue dots indicate proteins with decreased expression. The top 5 proteins with significantly different expression are shown. SAA1 Serum amyloid A1, SAA2 Serum amyloid A2, LCP1 Lymphocyte cytosolic protein 1, ORM2 Orosomucoid 2, TKT Transketolase. C Enrichment analysis based on biological processes and KEGG data. Fold enrichment is defined as the percentage of genes belonging to a pathway divided by the corresponding percentage of background. The size of the dots indicates the number of genes in the pathway. a and c Analyses are based on significantly up-regulated proteins. b and d Analyses are based on significantly down-regulated proteins. ARDS acute respiratory distress syndrome, ECM extracellular matrix, FDR false discovery rate, KEGG Kyoto Encyclopedia of Genes and Genomes, reg. regulation. [file 40560_2025_793_MOESM1_ESM.tiff]

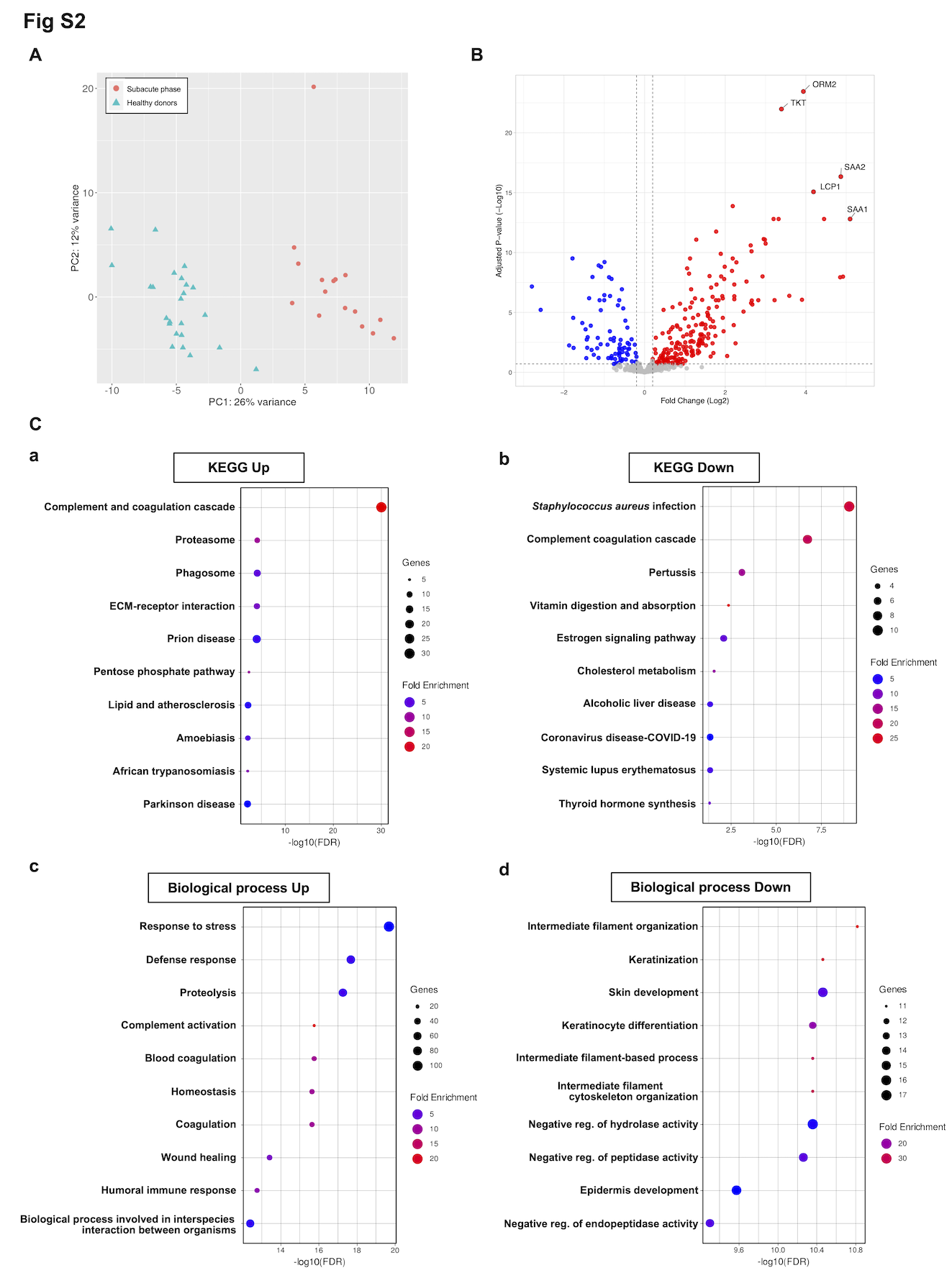

Supplement: Supplementary file 2 — Additional file 2: Figure S2. Comparison of subacute-phase plasma of ARDS patients with that of healthy donors. A Principal component analysis of plasma from ARDS patients in the subacute phase compared to plasma from healthy donors. Red dots indicate patients and light green triangles indicate the healthy donors. B Volcano plot of differences in plasma protein expression in the ARDS patients and healthy donors. The vertical dashed lines indicate a |log2 fold change|> 0.2. The horizontal dashed line indicates the threshold for FDR < 0.2. Red dots indicate proteins with increased expression, and blue dots indicate proteins with decreased expression. The top 5 proteins with significantly different expression are shown. SAA1 Serum amyloid A1, SAA2 Serum amyloid A2, LCP1 Lymphocyte cytosolic protein 1, ORM2 Orosomucoid 2, TKT Transketolase. C Enrichment analysis based on biological processes and KEGG data. Fold enrichment is defined as the percentage of genes belonging to a pathway divided by the corresponding percentage of background. The size of the dots indicates the number of genes in the pathway. a and c Analyses are based on significantly up-regulated proteins. b and d Analyses are based on significantly down-regulated proteins. ARDS acute respiratory distress syndrome, ECM extracellular matrix, FDR false discovery rate, KEGG Kyoto Encyclopedia of Genes and Genomes, reg. regulation. [file 40560_2025_793_MOESM2_ESM.tiff]

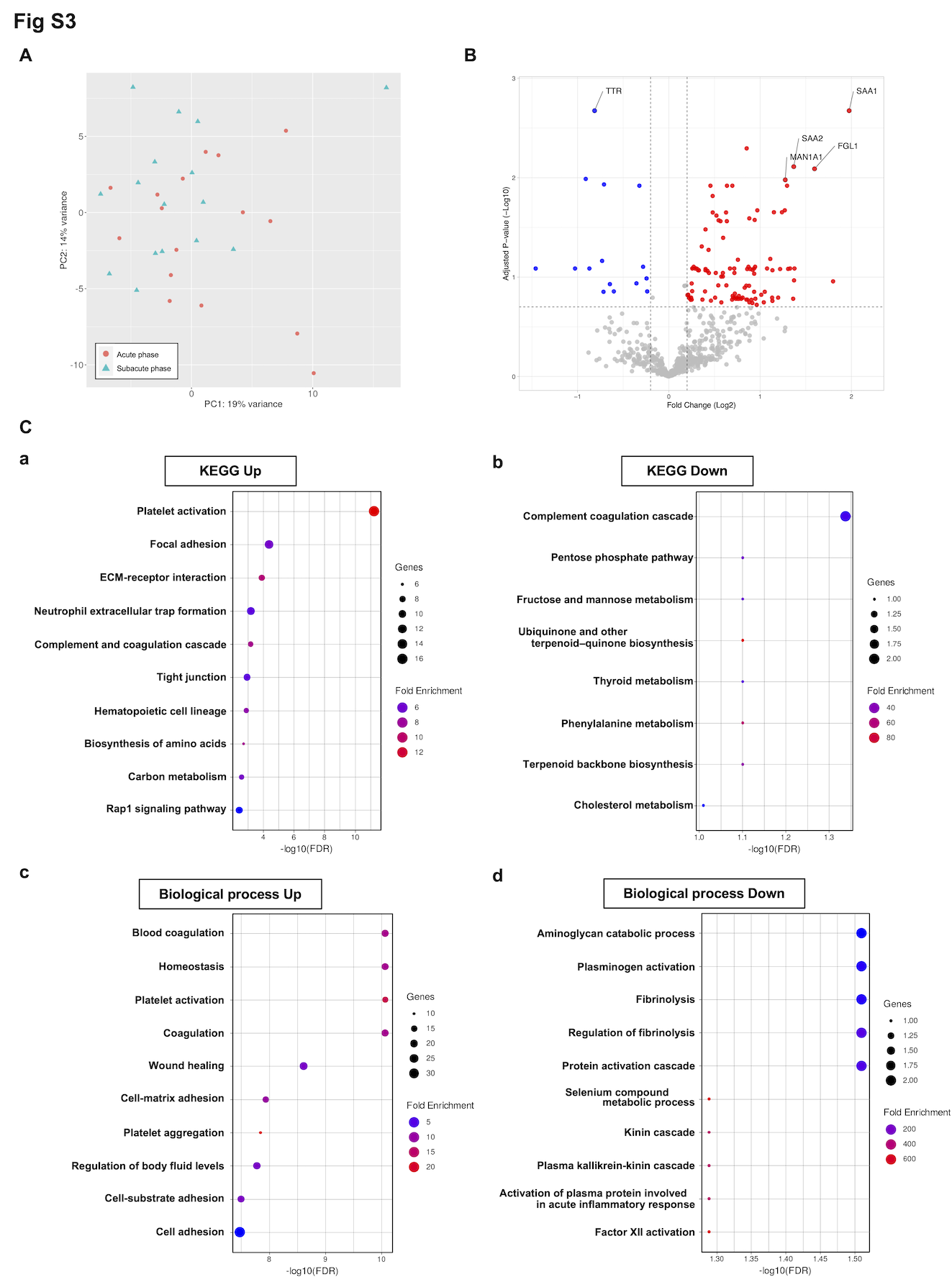

Supplement: Supplementary file 3 — Additional file 3: Figure S3. Comparison between acute and subacute plasma of patients with ARDS. A Principal component analysis of plasma from ARDS patients in the acute phase compared to that of the subacute phase. Red dots indicate patients and light green triangles indicate the healthy donors. B Volcano plot of the difference in plasma protein expression between acute and subacute plasma of ARDS patients. The vertical dashed lines indicate |log2 fold change|> 0.2. The horizontal dashed line indicates the threshold for FDR < 0.2. Red dots indicate proteins whose expression increased, and blue dots indicate proteins whose expression decreased. The top 5 proteins with significantly different expression are shown. SAA1 Serum amyloid A1, SAA2 Serum amyloid A2, FGL1 Fibrinogen-like protein 1, MAN1A1 Mannosidase Alpha Class 1A Member 1, TTR Transthyretin. C Enrichment analysis based on biological processes and KEGG data. Fold enrichment is defined as the percentage of genes belonging to a pathway divided by the corresponding percentage of background. The size of the dots indicates the number of genes in the pathway. a and c Analyses are based on significantly up-regulated proteins. b and d Analyses are based on significantly down-regulated proteins. ARDS acute respiratory distress syndrome, ECM extracellular matrix, FDR false discovery rate, KEGG Kyoto Encyclopedia of Genes and Genomes, reg. regulation. [file 40560_2025_793_MOESM3_ESM.tiff]

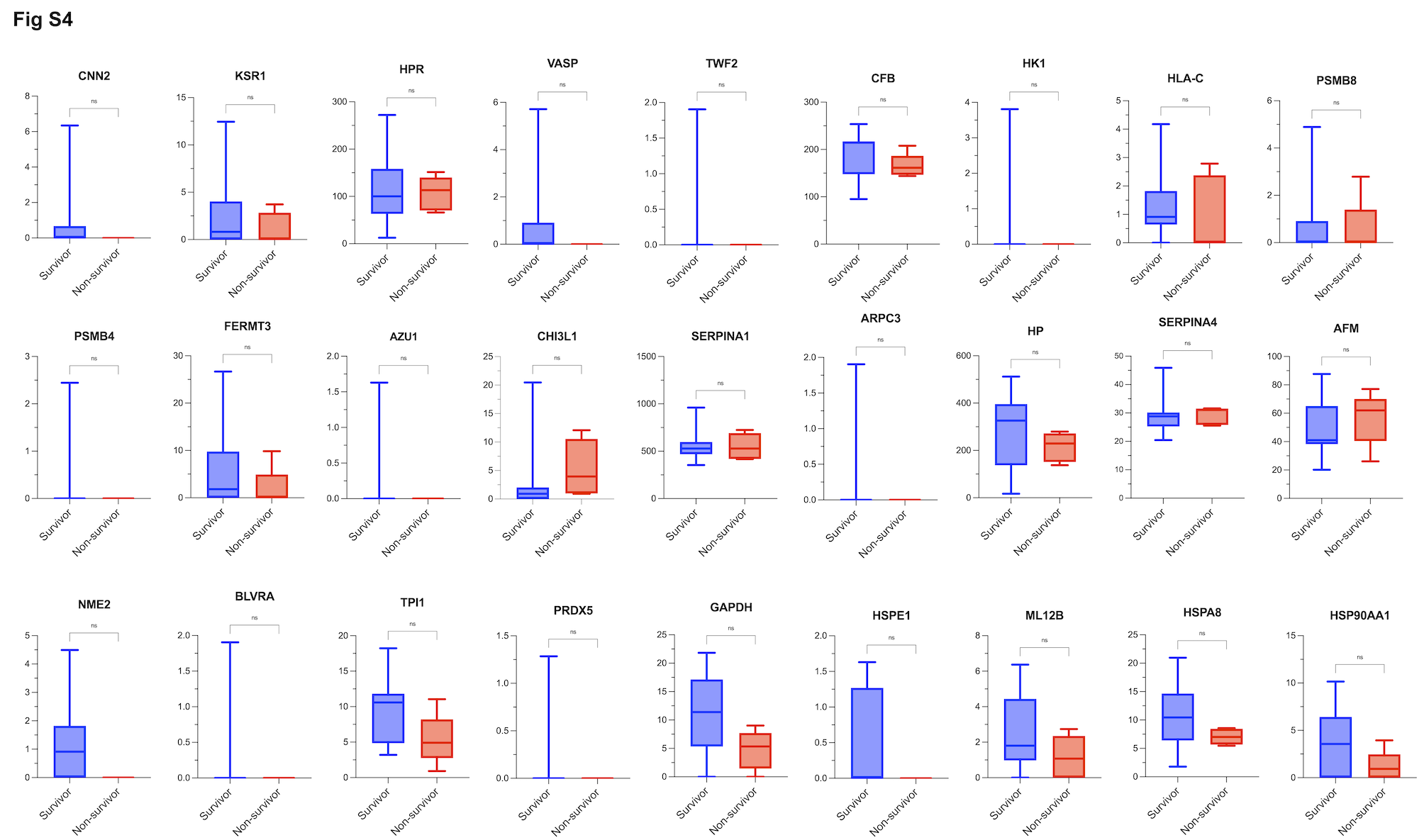

Supplement: Supplementary file 4 — Additional file 4: Figure S4. Comparison of each protein between survivors and non-survivors in acute-phase plasma. All values on the vertical axis for each protein are exponentially modified protein abundance index values. CNN2 Calponin 2, KSR1 Kinase Suppressor of Ras 1, HPR Haptoglobin-Related Protein, VASP Vasodilator-Stimulated Phosphoprotein, TWF2 Twinfilin Actin Binding Protein 2, CFB Complement Factor B, HK1 Hexokinase 1, HLA-C Major Histocompatibility Complex, Class I, C, PSMB8 Proteasome Subunit Beta 8, PSMB4 Proteasome Subunit Beta 4, FERMT3 Fermitin Family Member 3, AZU1 Azurocidin 1, CHI3L1 Chitinase 3-Like 1, SERPINA1 Serpin Family A Member 1, ARPC3 Actin-Related Protein 2/3 Complex Subunit 3, HP Haptoglobin, AZGP1 Alpha-2-Glycoprotein 1, Zinc-Binding, SERPINA4 Serpin Family A Member 4, AFM Afamin, NME2 Nucleoside Diphosphate Kinase B, BLVRA Biliverdin Reductase A, TPI1 Triosephosphate Isomerase 1, PRDX5 Peroxiredoxin 5, GAPDH Glyceraldehyde-3-Phosphate Dehydrogenase, HSPE1 Heat Shock Protein Family E (Hsp10) Member 1, ML12B Myosin Light Chain 12B, HSPA8 Heat Shock Protein Family A (Hsp70) Member 8, HSP90AA1 Heat Shock Protein 90 Alpha Family Class A Member 1. [file 40560_2025_793_MOESM4_ESM.tiff]

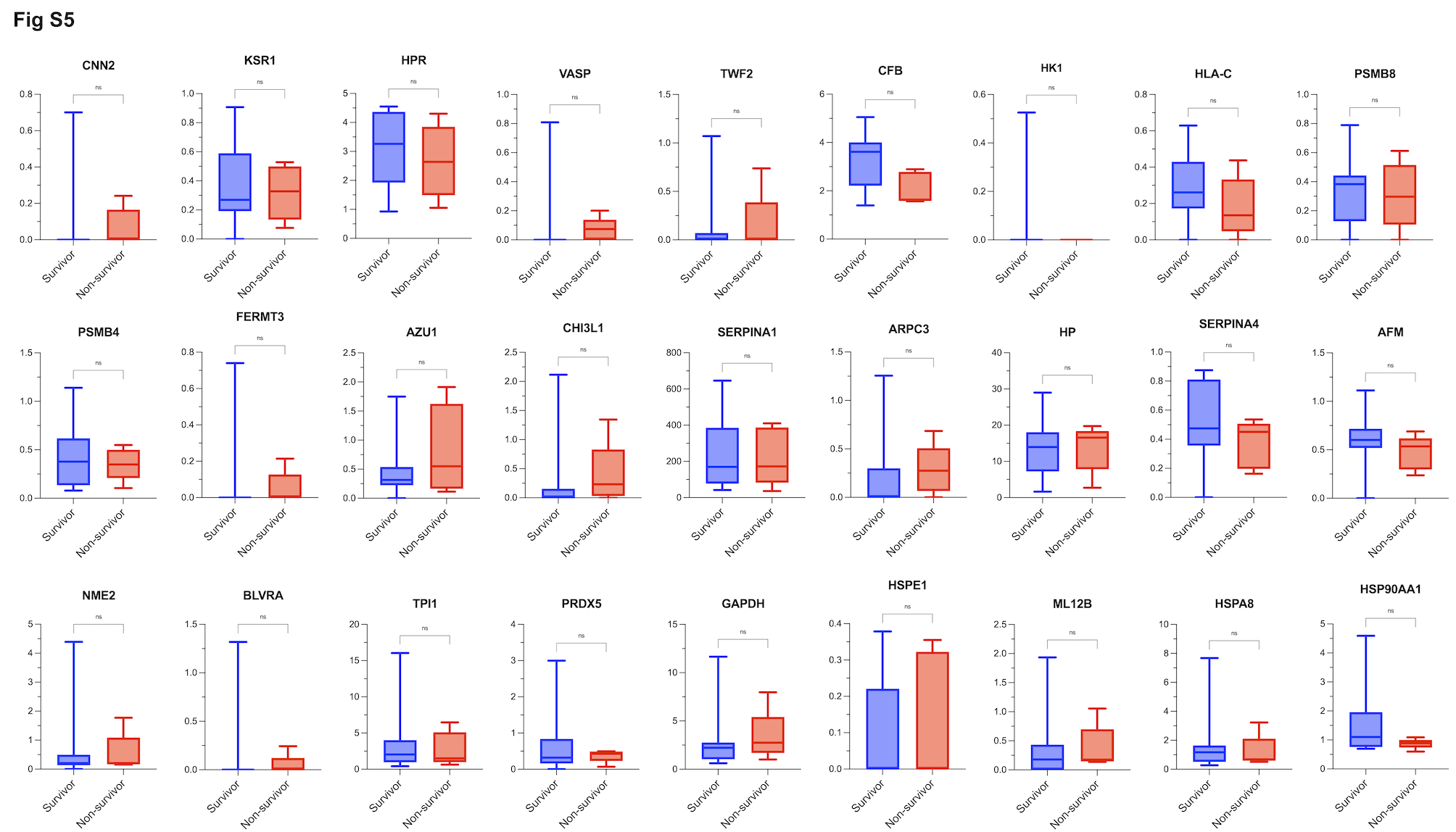

Supplement: Supplementary file 5 — Additional file 5: Figure S5. Comparison of each protein between survivors and non-survivors in acute-phase BALF. All values on the vertical axis for each protein are exponentially modified protein abundance index values. BALF bronchoalveolar lavage fluid, CNN2 Calponin 2, KSR1 Kinase Suppressor of Ras 1, HPR Haptoglobin-Related Protein, VASP Vasodilator-Stimulated Phosphoprotein, TWF2 Twinfilin Actin Binding Protein 2, CFB Complement Factor B, HK1 Hexokinase 1, HLA-C Major Histocompatibility Complex, Class I, C, PSMB8 Proteasome Subunit Beta 8, PSMB4 Proteasome Subunit Beta 4, FERMT3 Fermitin Family Member 3, AZU1 Azurocidin 1, CHI3L1 Chitinase 3-Like 1, SERPINA1 Serpin Family A Member 1, ARPC3 Actin-Related Protein 2/3 Complex Subunit 3, HP Haptoglobin, AZGP1 Alpha-2-Glycoprotein 1, Zinc-Binding, SERPINA4 Serpin Family A Member 4, AFM Afamin, NME2 Nucleoside Diphosphate Kinase B, BLVRA Biliverdin Reductase A, TPI1 Triosephosphate Isomerase 1, PRDX5 Peroxiredoxin 5, GAPDH Glyceraldehyde-3-Phosphate Dehydrogenase, HSPE1 Heat Shock Protein Family E (Hsp10) Member 1, ML12B Myosin Light Chain 12B, HSPA8 Heat Shock Protein Family A (Hsp70) Member 8, HSP90AA1 Heat Shock Protein 90 Alpha Family Class A Member 1. [file 40560_2025_793_MOESM5_ESM.tiff]

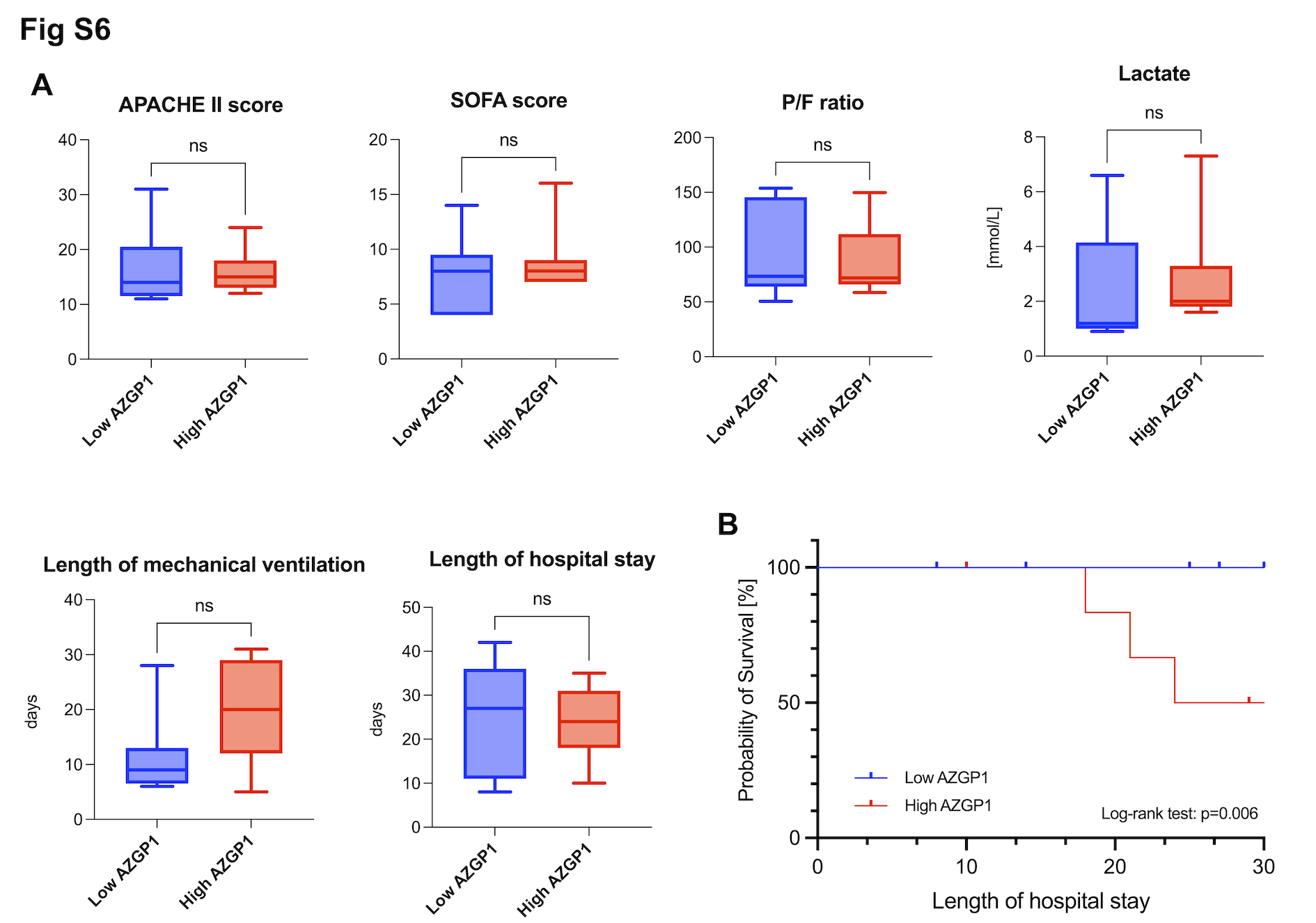

Supplement: Supplementary file 6 — Additional file 6: Figure S6. Comparison of clinical information in the low and high plasma AZGP1 groups. A Comparison of clinical information. Patients were divided into two groups using the plasma AZGP1 value at which the Youden index was maximal as the cutoff. APACHE Acute Physiology and Chronic Health Evaluation II, SOFA Sequential Organ Failure Assessment, P/F partial pressure of arterial oxygen/fraction of inspired oxygen. B The Kaplan–Meier curves for the two groups. The vertical axis shows the cumulative probability of survival, and the horizontal axis shows the days from admission to death. [file 40560_2025_793_MOESM6_ESM.tiff]
